# Could Tailored Chirp Stimuli Benefit Measurement of the Supra-threshold Auditory Brainstem Wave-I Response?

**DOI:** 10.1007/s10162-022-00848-0

**Published:** 2022-08-19

**Authors:** Jessica de Boer, Alexander Hardy, Katrin Krumbholz

**Affiliations:** 1 Hearing Sciences, School of Medicine, Mental Health & Clinical Neurosciences, University of Nottingham, Science Road, Nottingham, NG7 2RD UK; 2 Nottingham Biomedical Research Centre, Queens Medical Centre, Hearing Theme, Nottingham, NG7 2UH UK; 3 School of Psychology, University of Nottingham, University Park, Nottingham, NG7 2RD UK

**Keywords:** Cochlear dispersion, Optimized chirp stimulus, Synaptopathy, Hidden hearing loss, Auditory-evoked potentials, Objective audiology

## Abstract

Auditory brainstem responses (ABRs) to broadband clicks are strongly affected by dyssynchrony, or “latency dispersion”, of their frequency-specific cochlear contributions. Optimized chirp stimuli, designed to compensate for cochlear dispersion, can afford substantial increase in broadband ABR amplitudes, particularly for the prominent wave-V deflection. Reports on the smaller wave I, however, which may be useful for measuring cochlear synaptopathy, have been mixed. This study aimed to test previous claims that ABR latency dispersion differs between waves I and V, and between males and females, and thus that using wave- and/or sex-tailored chirps may provide more reliable wave-I benefit. Using the derived-band technique, we measured responses from frequency-restricted (one-octave-wide) cochlear regions to energy-matched click and chirp stimuli. The derived-band responses’ latencies were used to assess any wave- and/or sex-related dispersion differences across bands, and their amplitudes, to evaluate any within-band dispersion differences. Our results suggest that sex-related dispersion difference within the lowest-frequency cochlear regions (< 1 kHz), where dispersion is generally greatest, may be a predominant driver of the often-reported sex difference in broadband ABR amplitude. At the same time, they showed no systematic dispersion difference between waves I and V. Instead, they suggest that reduced chirp benefit on wave I may arise as a result of chirp-induced desynchronization of on- and off-frequency responses generated at the same cochlear places, and resultant reduction in response contributions from higher-frequency cochlear regions, to which wave I is thought to be particularly sensitive.

## Introduction

**Fig. 1:**
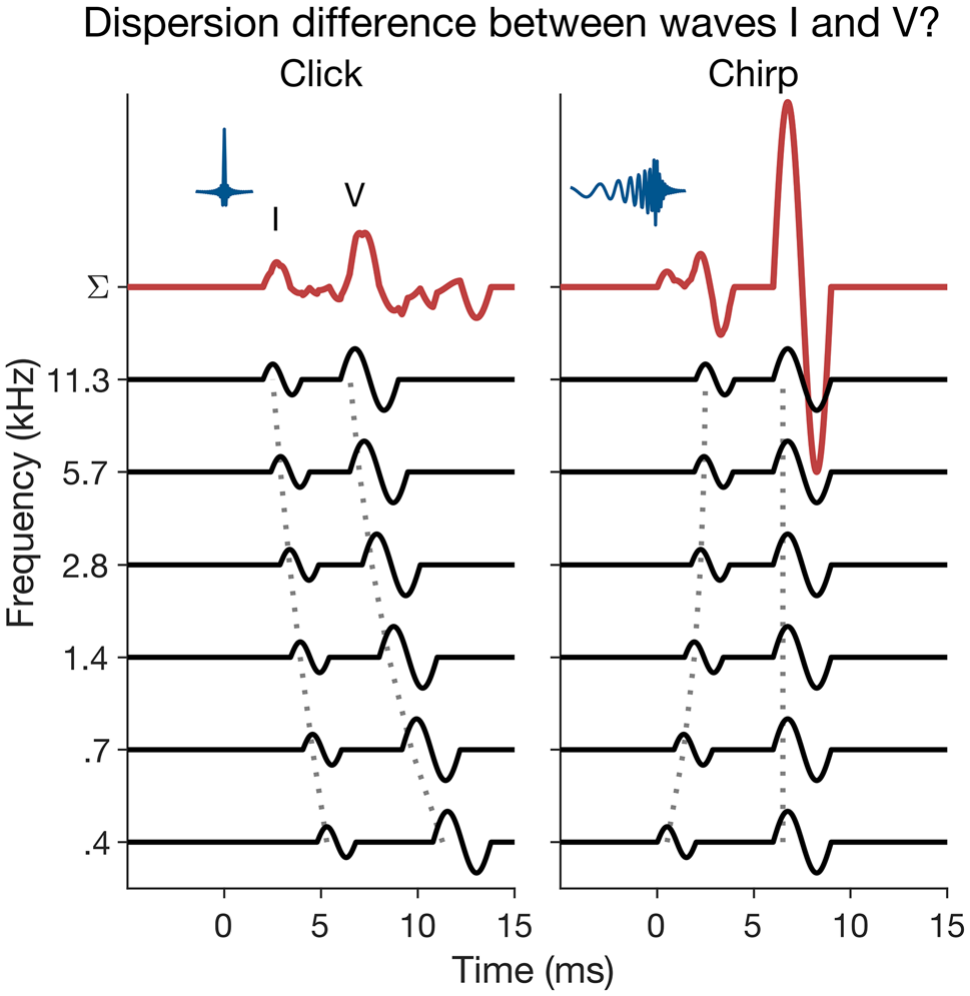
Schematic representation of click- (left) and chirp-evoked (right) ABRs, generated using 1-cylce sine waves to represent the derived-band wave-I and -V responses. Waves I and V were assumed to have different latency dispersions

Auditory brainstem responses (ABRs) are widely used for clinical hearing screening and objective hearing threshold estimation (Sininger 2007; British Society of Audiology 2019; The Joint Committee on Infant Hearing 2019). Clinical ABRs are mostly elicited by transient (very brief) sounds, particularly clicks and tone pips, and their evaluation is mostly limited to the wave-V deflection, the most prominent ABR wave, thought to be generated in the upper brainstem (e.g. Achor and Starr 1980; Møller and Jannetta 1983; Scherg and von Cramon 1985). Unsurprisingly then, some considerable research effort has been expended on finding optimal stimulus parameters to maximize the wave-V amplitude (Fobel and Dau 2004; Elberling et al. 2007, 2010; Elberling and Don 2010). The resulting stimuli were short chirps, designed to compensate for the cochlear travelling-wave delay (also referred to as “cochlear dispersion”), which causes ABR contributions from apical (lower-frequency) cochlear regions to be delayed relative to contributions from basal (higher-frequency) regions (Fig. 1). By advancing the apical response contributions, chirps increase the cross-frequency synchrony, and thus aggregate size, of the broadband wave-V response.

More recently, however, research focus has shifted towards the earlier wave-I deflection, which is thought to originate from the auditory nerve. This has been prompted by the suggestion, based on animal results, that the wave-I amplitude at supra-threshold sound levels may indicate the presence of cochlear “synaptopathy” (i.e. loss of primary afferent synapses; Kujawa and Liberman, 2009; Liberman and Kujawa 2017). Attempts at demonstrating synaptopathy in humans, however, have so far remained inconclusive (reviewed in Plack et al. 2016; Bramhall et al. 2019; Le Prell 2019). A major source of difficulty is the small size, and high degree of inter-individual variability, of the adult human wave I (Beattie 1988; Lauter and Loomis 1988; Lauter and Karzon 1990; Jiang et al. 1993).

Earlier findings, using highpass masking to isolate ABR contributions from frequency-restricted cochlear regions referred to as “derived bands” (Teas et al. 1962), have suggested that waves I and V exhibit the same or similar latency dispersion across frequencies (Don and Eggermont 1978; Eggermont and Don 1980; Ponton et al. 1992). This suggests that the existing wave-V-optimized chirps should equally benefit wave I. Few studies have tested this explicitly, and the existing — mostly indirect — evidence is mixed. Cebulla et al. (2014) measured newborn ABRs to clicks and wave V-optimized chirps and found a clear enhancement of the chirp-evoked wave-I amplitude. Similarly, Fobel and Dau (2004), in a study comparing different types of chirps, found that the optimal chirp for wave V also produced the “clearest wave I”. In contrast, Rodrigues and Lewis (2012), as well as Petoe et al. (2010) found a reduction or, in some cases, complete absence of wave I in the chirp-evoked responses. In a more recent study, Morimoto et al. (2019) systematically varied the time–frequency slope of the chirp stimulus and found that the optimal slope for wave I was steeper (corresponding to a more click-like stimulus) than that for wave V. They interpreted this finding as evidence that waves I and V exhibit different degrees of cochlear dispersion (as illustrated schematically in Fig. 1) — in apparent contradiction to the earlier findings based on derived-band latencies.

The optimal stimulus parameters that maximize ABR amplitudes may vary, not only between waves, but also between subjects. Don et al. (1993, 1994), for instance, reported differences in wave-V dispersion between males and females, with males showing a greater degree of dispersion than females. They speculated that this was due to males having longer cochleae, a proposition that has been supported by some anatomical studies (Sato et al. 1991; Thong et al. 2017), but questioned by others (Miller 2007; Osipov et al. 2013). If true, this would suggest that an optimal chirp would have to be steeper (more click-like) for females than for males.

**Fig. 2:**
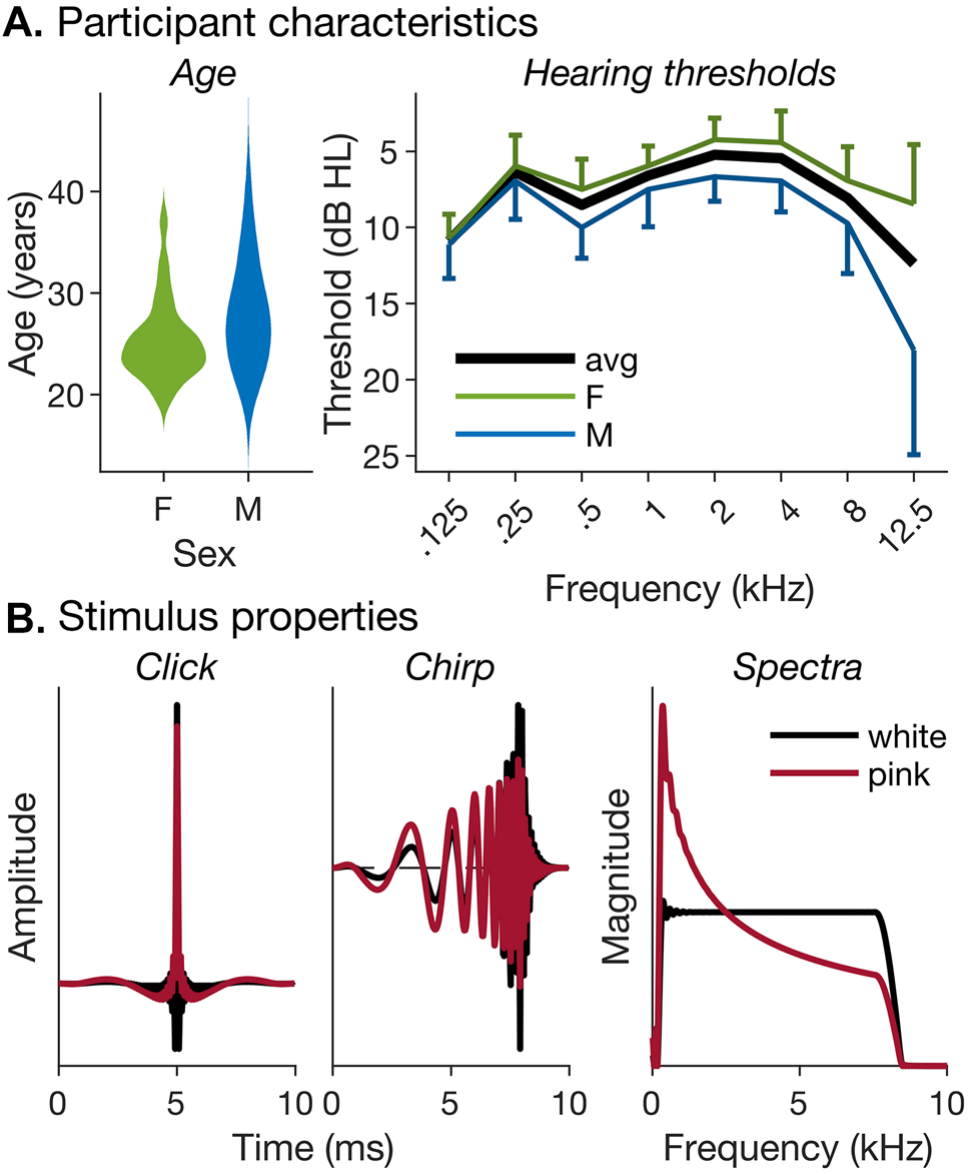
Participant and stimulus characteristics. **A** Left panel: violin plots of male (“M”) and female (“F”) age distributions, generated using kernel density estimation with optimal Gaussian kernel (Bowman and Azzalini 1997). Right panel: male, female and average (“avg”) hearing thresholds with 95% confidence intervals (*CI*s). **B** Waveforms (left and middle panels) and magnitude spectra (right panel) of pink and white click and chirp stimuli. Clicks and chirps had identical spectra and overall energy

This study aimed to test these previous claims of dispersion differences between waves I and V, and between males and females, and to establish whether, or to what degree, wave- and/or sex-tailored chirps could benefit supra-threshold wave-I measurements. Most previous studies concerned with the design of optimized chirp stimuli have used either broadband responses to chirps or derived-band responses to clicks. Here, we measured derived-band responses to both clicks and chirps. To ensure direct comparability, clicks and chirps were exactly matched for overall energy and spectral composition (see Fig. 2). Measuring derived-band responses to both clicks and chirps enabled us to assess any wave- and/or sex-related differences in latency dispersion across the derived frequency bands and also evaluate any chirp effects on response dispersion (or dyssynchrony) within bands. Our results suggest that a sex-related dispersion difference may exist within the most apical cochlear regions, where dispersion is greatest (see Fig. 1), but showed no systematic dispersion difference between waves I and V. Instead, they suggest an alternative explanation of the previous mixed results on wave-I chirp benefit, associated with the effect that chirps would be expected to have on the relative timing of on- and off-frequency responses arising from the same cochlear places.

## Methods

We used a derived-band ABR dataset comprising a total of 797 individual responses from 44 participants and up to 24 stimulus conditions. The data were combined from three separate experiments, which used different combinations of stimulus conditions, different numbers of highpass-masking conditions and different recording electrodes (see Table 1 and “ABR Acquisition”). Some participants took part in more than one experiment.

**Table 1:** Stimuli, highpass cutoff frequencies, electrode type and participant numbers used in the current experiments

	Stimuli	Highpass cutoff (kHz)	Electrode type	Subjects (F/M)
Experiment 1	White click and chirp Pink click and chirp	0.5, 1, 2, 4, 8 and Inf	Mastoid	22 (14/8)
Experiment 2	White click and chirp Pink chirp	0.5, 1, 2, 4, 8 and Inf	Tiptrode	9 (7/2)
Experiment 3	White chirp	1, 2, 4, 8 and Inf	Mastoid	26 (13/13)

### Participants

A total of 46 participants were initially recruited to the experiments included in this study. All participants first underwent pure-tone audiometric testing in both ears at frequencies of 0.125, 0.25, 0.5, 1, 2, 4, 8 and 12.5 kHz using a clinically calibrated Interacoustics AD629 audiometer (Middelfart, Denmark). Participants of Experiment 3 were additionally tested at frequencies between 14 and 20 kHz and completed a noise exposure history questionnaire, but these data were not analysed in this study. One participant did not continue on to the ABR measurements, because the participant’s hearing thresholds in the test ear exceeded 25 dB HL at conventional audiometric frequencies (i.e. between 0.25 and 8 kHz), and another participant was subsequently excluded because the participant failed to complete all highpass-masking conditions for any stimulus condition. The remaining 44 participants included 26 females and 18 males and had a mean age of 26.4 years [standard deviation = 4.56 years]. The average age difference between male and female participants was 2.69 years (Fig. 2A, left panel). An unpaired *t*-test showed the difference to be marginally significant [*t*(42) = 1.993, *p* = 0.053], but the associated Bayes factor analysis (see “Statistics”) suggested no evidence against the null hypothesis (of zero age difference; BF_10_ = 1.1). All included participants had hearing thresholds ≤ 25 dB HL at audiometric frequencies between 0.25 and 8 kHz in the test ear and none reported any history of audiological or neurological disease. Whilst there was some divergence between male and female hearing thresholds towards higher test frequencies (particularly above the conventional audiometric range, i.e. at 12.5 kHz; Fig. 2A, right panel), a linear mixed model (LLM) analysis (see “Statistics”) yielded no significant effects of sex (main effect: *χ*^2^(1) = 1.12, *p* = 0.291; sex-by-frequency interaction: *χ*^2^(7) = 11.0, *p* = 0.140) and the corresponding Bayes factor analyses indicated substantial or decisive evidence in favour of the null hypothesis (main effect: BF_01_ = 6.47; interaction: BF_01_ > 150). Participants were seated on a comfortable chair inside an electrically shielded, sound-attenuating booth (IAC Acoustics Company UK, Hampshire, UK) and gave prior written informed consent. Experimental procedures complied with the Declaration of Helsinki guidelines (Version 6, 2008) but were not formally pre-registered as set out in the Declaration’s 2014 amendment. They were approved by the Ethics Committee of the University of Nottingham Medical School.

### ABR Acquisition

ABRs were recorded at a sampling rate of 16.384 kHz using a BioSemi ActiveTwo system (BioSemi B.V., Amsterdam, Netherlands). The active electrodes were either flat-type Ag–AgCl mastoid electrodes or gold-wrapped tiptrodes, inserted in the ear canal. In both cases, the electrode signals were pre-amplified at the recording site, and the reference and ground electrodes were placed on the vertex and central forehead, respectively.

The evoking stimulus was either a click or a chirp and had either a flat spectral density profile (referred to as “white”) or a gradual low-pass profile with energy density at each frequency inversely proportional to the frequency value (referred to as 1/*f* or “pink”; see Fig. 2B). All stimuli were matched for overall energy and presented at a level corresponding to a peak-equivalent sound pressure level (SPL) of 90 dB for the white click. The chirp followed the CE chirp design developed by Elberling et al. (2007), which is based on click-evoked derived-band ABR latencies. In the CE chirp, different frequencies are staggered according to an exponential group delay function, ${t}_{g}=K\cdot{f}^{-D}$, where *t*_*g*_ is the group delay in milliseconds, *f* is frequency in kHz, and *D* and *K* are constants, equalling 0.436 and 4.54, respectively. All stimuli were generated by adding sinusoids at integer multiples of 10 Hz between 0.25 and 8 kHz. To avoid audible edge tones, the spectral edges were rounded using quarter-sine or -cosine ramps with a width corresponding to the relevant normal equivalent rectangular auditory filter bandwidth (ERBN; Glasberg and Moore 1990).

Both the click and chirp stimuli were presented either in quiet (referred to as “broadband” condition), or in a background of highpass-filtered noise intended to mask any response contributions from above the highpass cutoff frequency (referred to as “highpass-masked” conditions). Either four or five different cutoff frequencies were used in different experiments, ranging from 0.5 or 1 to 8 kHz in octave steps (Table 1). Each highpass noise was filtered from a broadband noise with the same spectral profile as the to-be-masked stimulus (white or pink) and an overall level of 80 dB SPL. Filtering was performed in the frequency domain using a brick-wall filter design to create a 10.5-s cyclical buffer.

Different stimuli and masking conditions were measured in separate experimental runs, which lasted between 4 and 5 min each and were presented in a randomized order across participants. When used, a fresh highpass noise buffer was created prior to the run start and played out continuously during the entire run duration. The relevant stimulus was presented at a rate of 20/s and with alternating polarity. A run was terminated when the requisite number of stimuli had been presented (6000 in Experiments 1 and 2, 5000 in Experiment 3).

All stimuli were generated digitally at a 50-kHz sampling rate using Matlab (The Mathworks, Natick, MA, USA), and digital-to-analogue converted with a 24-bit amplitude resolution using a Tucker Davis Technologies (Alachua, FL, USA) real-time signal processor (RP2.1 with HB7 headphone buffer in Experiments 1 and 2, RZ6 multi-I/O processor in Experiment 3). They were presented monaurally to the left ear through ER-2 insert earphones (Etymotic Research Inc., Elk Grove Village, IL, USA).

### ABR Analysis

All ABR processing was performed in Matlab. First, the data were low-pass filtered at 2 kHz and highpass filtered at either 100 or 150 Hz (both the low- and highpass filters were implemented as 4th-order Butterworth IIR filters). The 150- and 100-Hz highpass frequencies were chosen to maximize the sizes of waves I and V, respectively. Then, the data were divided into 45-ms epochs including a 5-ms pre-stimulus baseline and submitted to a Bayesian weighted averaging procedure (Elberling and Wahlgreen 1985). The resulting average responses were cross-spliced between their 100- and 150-Hz-filtered versions using 2-ms linear ramps centred on 5 ms to create a single average ABR for each participant and stimulus condition. The resulting cross-spliced responses contained the 150-Hz-filtered version in the wave-I time range (≤ 4 ms) and the 100-Hz-filtered version in the wave-V time range (≥ 6 ms).

The highpass-masked responses for successive cutoff frequencies [(0.5,) 1, 2, 4 and 8 kHz] were subtracted to isolate response contributions from the intervening frequency regions [i.e. (0.5–1,) 1–2, 2–4 or 4–8 kHz]. The resulting difference responses are commonly referred to as “derived-band responses”. In addition, the highpass-masked response for the lowest cutoff frequency (0.5 or 1 kHz) was taken to represent response contributions from below that cutoff frequency (i.e. ≤ 0.5/1 kHz), and the response for highest cutoff frequency (8 kHz) was subtracted from that for the broadband condition to represent response contributions from above 8 kHz (≥ 8 kHz), and thus above the stimulus frequency range. In line with common convention, the derived-band responses are referred to by their octave-spaced centre frequencies (0.4, 0.7, 1.4, 2.8, 5.7 and 11.3 kHz), with 0.4 representing the ≤ 0.5-kHz band, and 11.3 the ≥ 8-kHz band.

**Fig. 3:**
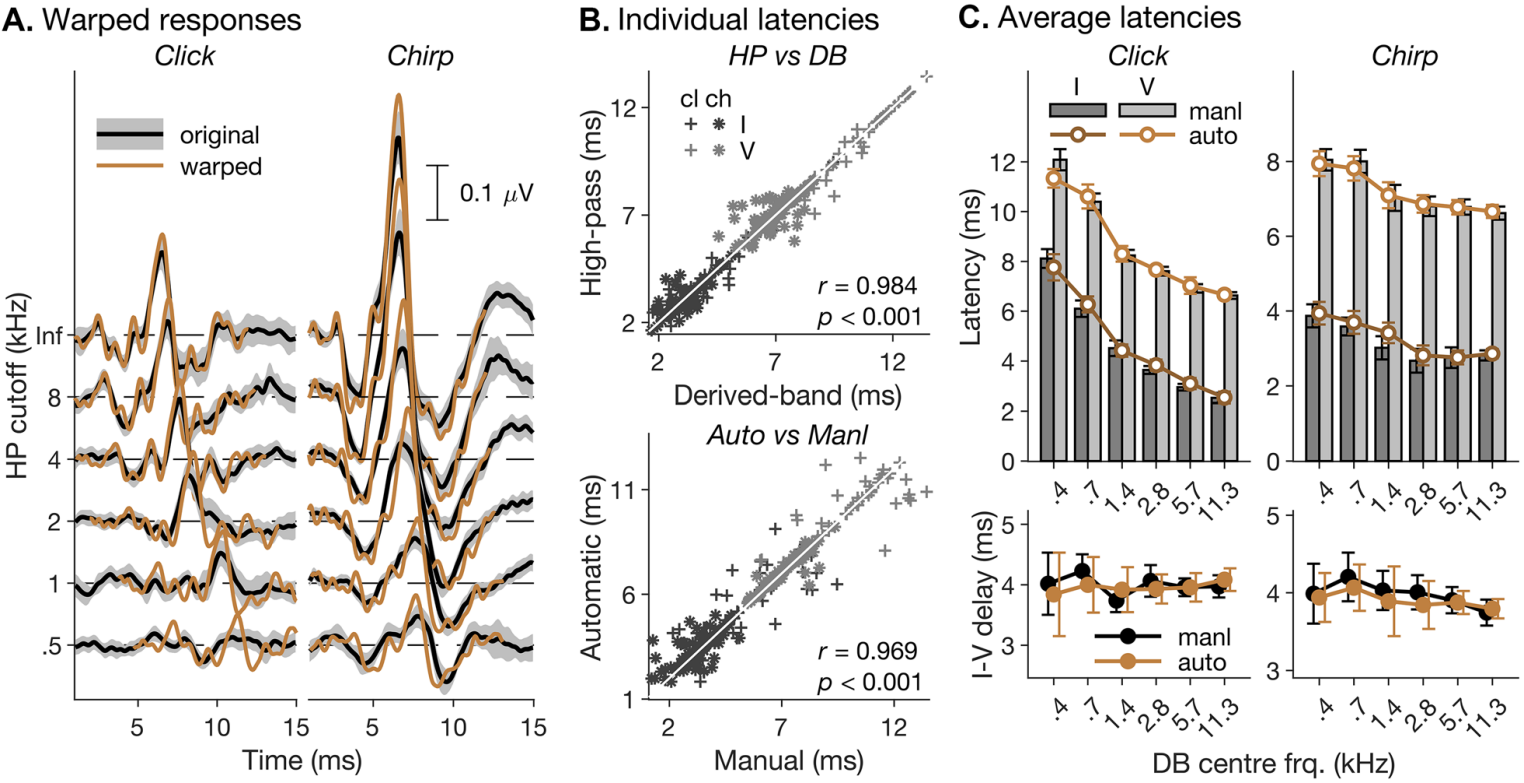
Evaluation of automatic peak picking procedure. **A** Comparison of cross-sectional average (“original”) and structural average (“warped”) highpass-masked responses to the white click and chirp stimuli (left and right panels). Responses for different highpass-masking cutoff frequencies (indicated on the ordinate) are staggered vertically for clarity. The grey patches indicate 95% *CI*s of the cross-sectional average responses. **B** Top panel: scatter plot of individual manually picked derived-band (abscissa) and highpass-masked latencies across both stimulus conditions (“cl” = click, “ch” = chirp) and both waves (I and V). The identity line is indicated in white. Bottom panel: same as in top panel, but for the manually and automatically picked highpass-masked latencies. Insets show Pearson correlation coefficients and associated *p*-values. **C** Top panels: average manually (bars) and automatically picked (symbols and lines) wave-I and -V latencies for the white click- and chirp-evoked responses from Experiment 1. Error bars show 95% *CI*s. Lower panels: average manually and automatically picked wave-I-to-V inter-peak latency differences (referred to as “I-V delay”)

ABR wave latencies and amplitudes are usually determined by finding the relevant wave within each individual response and manually picking its peak and subsequent trough. Usually, this process is repeated by multiple observers to avoid bias or error. However, given that the current dataset comprised nearly 800 individual responses, and each response had to be picked for not only one, but two waves (I and V), manual picking was not a viable option. Given the shortcomings of earlier automated picking procedures, such as reliance on potentially unrealistic simplifying assumptions of response shape invariance (Elberling 1979; Vannier et al. 2002; Valderrama et al. 2014), or potential creation of selection bias as a result of peak/trough search limitation to restricted time windows (Guest et al. 2017; Prendergast et al. 2017), we opted for a more recent procedure specifically developed to overcome these shortcomings (Krumbholz et al. 2020). The procedure uses non-linear curve registration (also referred to as “dynamic time warping”, Wang and Gasser 1997) to temporally align the individual responses (Ramsay and Li 1998) and create a structural average response, which minimizes temporal blurring as a result of inter-individual latency variability (Kneip and Gasser 1992; see Fig. 3A for a comparison between structural and cross-sectional average responses for the white click and chirp stimuli). The structural average response is used to pick the relevant waves’ peaks and troughs and the corresponding individual peak and trough latencies are then derived by applying the inverse of the individual registration, or time-warping, functions. Temporal alignment was performed using the “average-target” (*at*) warping procedure and the penalized squared difference of the derivatives (PSDD) fitting criterion (see Krumbholz et al. 2020 for details). The cross-sectional average broadband response for the relevant stimulus condition was used as initial warping target.

Given that non-linear curve registration, like manual peak picking, is susceptible to noise and will ultimately fail when the signal-to-noise ratio (SNR) becomes too small (Krumbholz et al. 2020), and given that derived bands tend to have poor SNRs, particularly for the lower-frequency bands (Don and Eggermont 1978), we opted to use the latencies of the highpass-masked responses as a proxy for the derived-band latencies. Given that the earliest response contributions to the highpass-masked responses must arise from the highest included frequency regions, highpass-masked and derived-band latencies should be highly similar. The scatter plot in Fig. 3B (top) confirms that this was indeed the case: manually picked highpass-masked and derived-band latencies for the white click and chirp stimuli from Experiment 1 showed a high and significant correlation (*r* = 0.984, *p* < 0.001). An LMM analysis showed no significant effect of response type (highpass-masked vs derived-band; *χ*^2^(2) = 0.635, *p* = 0.728), and the associated Bayes factor analysis yielded decisive evidence in favour of the null hypothesis (BF_01_ > 150).

To validate the automatic picking procedure, we compared a subset of the automatically picked latencies (involving the responses to the white click and chirp stimuli from Experiment 1) with corresponding manually picked latencies (Fig. 3B and C bottom). The manual picking was performed by, and cross-validated between, three experienced, blinded observers (authors JdB, AH and KK). Whilst, for the absolute wave-I and -V latencies (Fig. 3C, top), the effect of picking method did manage to reach significance in an LMM analysis (*χ*^2^(6) = 24.2, *p* < 0.001), the associated Bayes-factor analysis suggested decisive evidence in favour of the null hypothesis (that manually and automatically picked latencies were equal; BF_01_ > 150). For the interpeak latency difference between waves I and V (referred to as I–V delay; Fig. 3C, bottom), neither the LMM nor the Bayes-factor analysis suggested that the manually and automatically picked latencies were different from one another (*χ*^2^(2) = 0.674, *p* = 0.714; BF_01_ > 150).

“Stacked ABRs” are created by temporally aligning the derived-band responses through linear time-shifts with appropriate shift delays (e.g. corresponding to the reverse of the wave-I or -V latencies), and then summing the aligned responses across bands (Don et al. 1997). Like chirps, stacked ABRs aim to compensate for cross-frequency latency dispersion. When compensating for sex-specific or individual latency dispersion (see “Simulated Responses to Wave- and/or Sex-Optimized Chirps Using Stacked ABRs and Individual Stacking Significantly Increases the Effective Wave-I Amplitude”), alignment was first performed separately for the wave-I and -V latencies, and the resulting wave-I- and wave-V-stacked responses were then cross-spliced (see above), so that the wave-I time range (≤ 4 ms) contained the wave-I-stacked response and the wave-V time range (≥ 6 ms) contained the wave-V-stacked response.

### Statistics

Statistical analyses were conducted in *R* (R Core Team 2013) and in Matlab. Linear and non-linear mixed-effects models (LMMs and NLMMs) were implemented using the *lme* and *nlme* functions of the *nlme* package for *R* (Pinheiro et al. 2021). Both LMMS and NLMMs included appropriate fixed effects and random by-participant intercepts. Model parameters were fitted using likelihood maximization (“ML” option). In the LMMs, fixed effects included, where appropriate, sex (F/M), wave (I/V), stimulus (click/chirp), spectral condition (white/pink) and derived-band frequency [0.4, 0.7, 1.4, 2.8, 5.7, 11.3 kHz], all of which were treated as factors. The NLMMs included a fixed dispersion effect, which was modelled using the same exponential dispersion function as used for creating the chirp stimulus (${t}_{g}=K\cdot {f}^{-D}$), but in this case, with variable *K* and *D* (see “Results”). In the NLMMs, derived-band frequency was treated as a continuous variable, with values corresponding to the band’s upper edge frequencies. The frequency value for the ≥ 8-kHz band (where the upper edge frequency was infinite) was set to 16 kHz, an octave above the lower edge frequency (8 kHz). In both scenarios (LMMs and NLMMs), alternative and null models (that either did or did not include the effect of interest) were constructed using a top-down model building strategy — starting from all possible effects and effect interactions and removing those that did not significantly improve the model fit (according to marginal *f*-tests). The resulting models were then compared with a chi-squared likelihood-ratio test to yield a frequentist *p*-value, and their respective Bayesian information criteria (BIC) were used to calculate associated Bayes factors (BFs; Wagenmakers 2007; Jarosz and Wiley 2014). BIC penalize increase in the number of model parameters more stringently than Akaike information criteria (AIC), which, in turn, penalize more stringently than likelihood ratios. Depending on which model carried more evidence, BFs are either reported as the ratio of evidence for the null over the alternative model (referred as BF_01_) or for the alternative over the null model (BF_10_).

Model residuals were tested for heteroscedasticity using Levene’s test (*leveneTest* of the *car* package; Fox and Weisberg 2020) and inspected for normality using quantile–quantile plots. Where heteroscedasticity was significant, models were refitted after inverse-variance-weighting the data for each factor level. No significant normality violations were observed in the current data.

Stacked and derived-band ABR waveforms were compared pointwise after first aligning the to-be-compared waveforms in time using a similar non-linear curve registration procedure as used for automatic extraction of the wave latencies (see above). This prevents confounding amplitude and latency differences between corresponding waveform features (Huang and Jansen 1985; Gupta et al. 1996; Karamzadeh et al. 2013). As for automatic latency extraction, alignment was applied to individual participants’ waveforms using the *at* warping procedure and *PSDD* fitting criterion. The initial warping target, in this case, was the average of the cross-sectional averages of the to-be-compared responses (in the case of the derived bands, alignment was performed separately for each band). After alignment, stacked ABRs were compared with pointwise *t*_*max*_ permutation tests (Blair and Karniski 1993; Westfall et al. 1993) implemented in Matlab by Groppe et al. (2011). The tests were applied to the time range from 0 to 12 ms, using *n* = 5000 permutations and a family-wise type-I error rate of $\alpha$ = 0.05. Derived-band ABR amplitudes were compared by integrating the relevant responses’ pointwise absolute values over a time window spanning from the start to the end of either wave I (2–5 ms) or wave V (5–9 ms) separately, or both together (2–9 ms). The resulting aggregate absolute responses were compared with LMMs and planned comparisons using two-sample *t*-tests allowing for unequal variances.

## Results

Using a collated set of derived-band ABR data from three separate experiments, we here examine whether appropriately tailored chirp stimuli could benefit measurement of the supra-threshold ABR wave I, which is especially small and inter-individually variable. The stimuli were either clicks or CE chirps (Elberling et al. 2007), with either flat (referred to as “white”) or 1/*f*-weighted (referred to as “pink”) spectral profiles (see “ABR Acquisition”). The motivation for including pink stimuli was to test whether pink stimuli, which contain more energy at lower frequencies, where dispersion is greater, are associated with greater chirp benefit than white stimuli.

First, we assess the derived-band latencies of waves I and V for any wave- or sex-dependent differences in across-band latency dispersion. Then, we simulate the effects of using wave- and/or sex-tailored chirps more directly using “stacked ABRs”, which, like chirps, aim to increase response synchronization by compensating for latency dispersion (Don et al. 1997, 2005, 2009). Finally, we evaluate the derived-band amplitudes, which reflect the degree of response dyssynchrony, or dispersion, within each derived frequency band.

Our results revealed two findings that were unexpected at the study outset. The first, reported in the upcoming section (“Chirp-Evoked ABRs Exhibit Greater Underlying Dispersion than Click-Evoked ABRs”), was that the difference in latency dispersion between the click- and chirp-evoked responses did not equal the dispersion (or, group-delay function; see “ABR Acquisition”) of the chirp stimulus, as would be expected if click- and chirp-evoked responses were related linearly to one another. The second, reported in the final “Results” section (“Chirps Can Cause Substantial Reduction in Higher-Frequency Derived-Band ABR Amplitudes”), was that higher-frequency chirp-evoked derived-band responses were in some cases substantially reduced compared to the corresponding click-evoked responses, particularly in conditions were the stimulus spectral profile was pink rather than white.

### Chirp-Evoked ABRs Exhibit Greater Underlying Dispersion than Click-Evoked ABRs

**Fig. 4:**
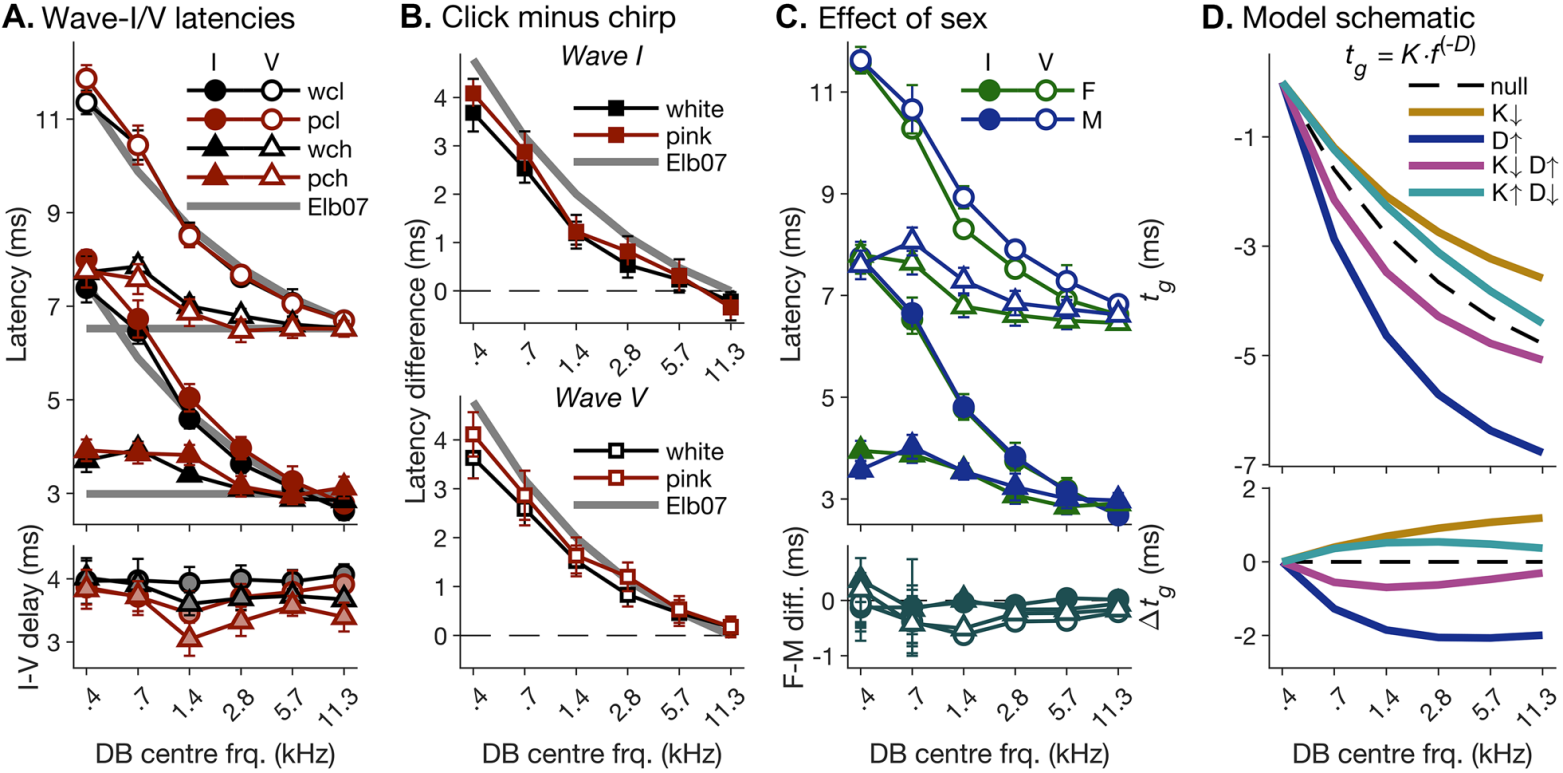
Frequency-dependence of derived-band latencies. **A** Average derived-band latencies for waves I and V, plotted as a function of the corresponding band centre frequencies (see “ABR Acquisition”). Waves I and V are indicated by different marker face colours; click and chirp stimuli are indicated by different marker shapes, and pink and white stimulus spectral profiles are indicated by different line colours. The top panel shows the absolute latencies, and the bottom panel the associated I–V delays (averages and 95% *CI*s). The bold grey lines show latency predictions based on the group delay function used for creating the chirp stimulus (Elberling et al. 2007 see “ABR Acquisition”). If click- and chirp-evoked responses showed the same underlying dispersion, the chirp latencies would be expected to vary minimally across bands. **B** Differences between the click- and chirp-evoked response latencies shown in **A**. The top and bottom panels show the wave-I and -V results, respectively. **C** Average wave-I and -V latencies of male (“M”) and female (“F”) participants, plotted in the same way as in **A**. For clarity, latencies were averaged across white and pink spectral conditions. **D** Schematic effects of changing the dispersion parameters, *K* and *D*, on the exponential group delay function, ${t}_{g}=K\cdot {f}^{-D}$, used to test the hypothesis that dispersion differed between waves I and V, or between males and females. The functions are plotted in the same way as the measured latencies in **A** and **C**. For clarity, the functions were normalized by subtracting the group delay for the lowest-frequency (0.4 kHz) derived band

The top panel in Fig. 4A shows the derived-band latencies of the wave-I and -V peaks (for which corresponding highpass-masked latencies served as proxy; see “ABR Analysis”) for all tested stimulus conditions (white/pink click/chirp), plotted against the band centre frequencies. The click-evoked latencies showed a strong degree of cross-band dispersion, decreasing steeply with increasing centre frequency. This was expected, because, due to the cochlear travelling-wave delay, response contributions from basal (higher-frequency) cochlear regions are associated with shorter latencies (see Fig. 1, left panel, for a schematic illustration). The chirp-evoked latencies, by contrast, showed a much lesser degree of dispersion. Again, this was expected, as chirps are designed to cancel the effect of the travelling-wave delay.

Figure 4A also shows that the click-evoked latencies accorded well with the group-delay function of the chirp stimulus, as would be expected given that the function is based on click-evoked derived-band latencies (Elberling et al. 2007). Based on the assumption that frequency-specific contributions to click- and chirp-evoked responses are merely time-shifted relative to one another, but otherwise equal (e.g. Don et al. 2009), the chirp-evoked latencies would be expected to show minimal remaining dispersion. This, however, was not the case. Instead, the chirp-evoked latencies showed consistent residual dispersion across both waves (I and V) and both spectral conditions (white/pink), suggesting a discrepancy in underlying dispersion between clicks and chirps.

This is further illustrated in Fig. 4B, which shows the difference between the click- and chirp-evoked latencies in direct comparison to the group-delay function of the chirp. If the effect of the chirp had been to merely time-shift the derived-band responses, the latency difference should roughly equal the group-delay function. Instead, it was consistently shallower. To test this discrepancy statistically, we fitted the click- and chirp-evoked latencies with a linear mixed-effects model (LMM) after first adjusting the click-evoked latencies by subtracting the group-delay function of the chirp. A frequentist likelihood ratio test between the null and alternative models (see “Statistics”) showed a significant stimulus-by-frequency interaction (*χ*^2^(5) = 102, *p* < 0.001), and the associated Bayes factor analysis suggested decisive evidence in favour of the alternative hypothesis (BF_10_ > 150). Figure 4B suggests that the dispersion discrepancy between click and chirp responses was greater for the white than pink stimuli. However, whilst this difference was consistent between waves I and V (compare top and bottom panels), it failed to reach statistical significance (*χ*^2^(5) = 7.73, *p* = 0.172; BF_01_ > 150).

### Does ABR Latency Dispersion Differ Between Waves I and V, or Between Males and Females?

According to the results of Morimoto et al. (2019), wave I would be expected to exhibit less latency dispersion (smaller latency differences across derived bands) than wave V (see Fig. 1 for a schematic depiction). As a result, the waves’ inter-peak latency difference, referred to as “I-V delay” (Fig. 4A, bottom panel), should be greatest at the lowest derived-band frequency and then decrease monotonically towards higher frequencies. Conversely, if waves I and V exhibited the same or similar dispersion, the I–V delay should be constant across frequencies. Neither scenario seems to fully match the current data: whilst, for some stimulus conditions (white click and, to a lesser degree, white chirp), the I–V delay was indeed largely constant; for others (pink chirp and, to a lesser degree, pink click), it showed considerable variation across frequencies, which was, however, not monotonic, but rather increased first and then decreased with increasing frequency.

Similarly, if, as suggested by Don et al. (1993, 1994), cochlear dispersion was greater in males than females (due to greater cochlear length), derived-band latencies should show greater dispersion for males compared to females, and this should be true for both waves I and V. In the current data, male and female latencies were nearly identical for wave I (Fig. 4C, top panel) and, whilst they showed a clear difference for wave V, the difference, again, did not decrease monotonically with increasing frequency (Fig. 4C, bottom panel).

To investigate whether the observed wave- or sex-related differences in derived-band latencies were compatible with a difference in cochlear dispersion, we fitted the individual latencies with a non-linear mixed-effects model (NLMM; see “Statistics”), which estimated each latency as the sum of a constant offset, *A*, plus a frequency-dependent group-delay, modelled by the same type of exponential function as used to create the chirp stimulus (${t}_{g}=K\cdot {f}^{-D}$). To enable concurrent fitting of both the click- and chirp-evoked latencies with the same group-delay function, we adjusted the chirp-evoked latencies by adding the average click- minus chirp-evoked latency differences across all participants, waves and spectral profiles before fitting. In the alternative model, both the constant offset, *A*, and the dispersion constants, *K* and *D*, were allowed to vary across sexes, waves, stimuli (click/chirp) and spectral profiles (white/pink), whilst, in the null model, only *A* was allowed to vary. Allowing both *K* and *D* to vary concurrently — and thus potentially oppositely — to one another enabled the alternative model to fit dispersion functions with greater or lesser curvature, but similar overall dispersion, and thus match the non-monotonic frequency dependence observed in the wave- and sex-related latency differences (see Fig. 4D for schematic examples).

**Fig. 5:**
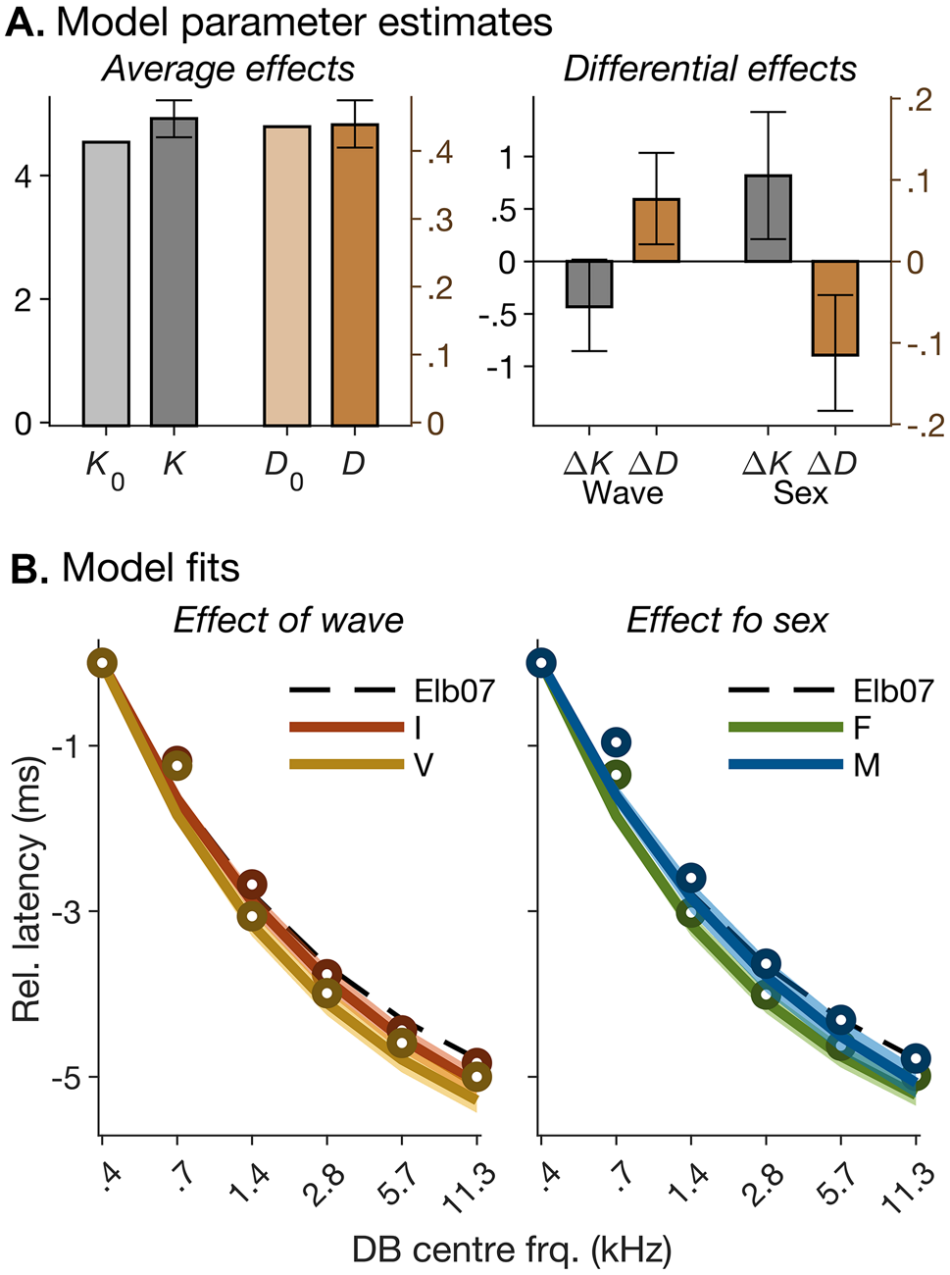
Summary of non-linear mixed-effects model (NLMM) fits of the derived-band latencies shown in Fig. 4. **A** Left panel: best-fitting average dispersion parameters, *K* and *D*, across waves and sexes. The *K* values refer to the left ordinate, and *D* values, to the right ordinate. For comparison, the lighter-shaded bars show the values, *K*_0_ = 4.54 and *D*_0_ = 0.436, used for creating the chirp stimulus. Right panel: linear differences, Δ*K* and Δ*D*, in *K* and *D* between waves (left; I–V) and sexes (right; F–M). As in the left panel, Δ*K* and Δ*D* values refer to the left and right ordinates, respectively. **B** Average best-fitting group-delay functions for waves I and V (left) and for males and females (right). For comparison, the average measured data (symbols) and the group delay function used to create the chirp stimulus (dashed lines labelled “Elb07”) are also shown. The semi-transparent patches show the 95% *CI* (based on multivariate normal simulations using the model variance–covariance structure). As in Fig. 4D, the functions and measured latencies were normalized by subtracting the value for the lowest-frequency (0.4 kHz) derived band

A likelihood ratio comparison showed that an alternative model in which the dispersion parameters, *K* and *D*, depended linearly and independently on both wave and sex created a significantly better fit than the null model (*χ*^2^(4) = 20.9, *p* < 0.001). The associated Bayes factor analysis, however, yielded very strong evidence in favour of the null hypothesis (that *K* and *D* were constant across waves and sexes; BF_01_ = 47.7). The left panel in Fig. 5A shows that the best-fitting intercepts in *K* and *D* were close to the values used for creating the CE chirp (*K* = 4.54, *D* = 0.436; Elberling et al. 2007). The right panel in Fig. 5A confirms that the best-fitting differential effects of wave and sex on *K* and *D* (Δ*K* and Δ*D*) had opposite signs, thus creating opposite effects on the fitted dispersion slopes. As a result, whilst each effect on its own was relatively large — ranging from 8.4% for the wave effect on *K* (leftmost bar in Fig. 5A, right panel) to 25% for the sex effect on *D* (rightmost bar in the same panel), the overall effects of wave and sex on the fitted dispersion slopes were relatively small (Fig. 5B).

The most significant effect revealed by the NLMM analysis was a sex-by-wave interaction in the constant offset, *A*, which models a constant sex difference in the wave-V, but not wave-I latencies (*χ*^2^(4) = 21.2, *p* < 0.001; BF_10_ > 150).

### Simulated Responses to Wave- and/or Sex-Optimized Chirps Using Stacked ABRs

**Fig. 6:**
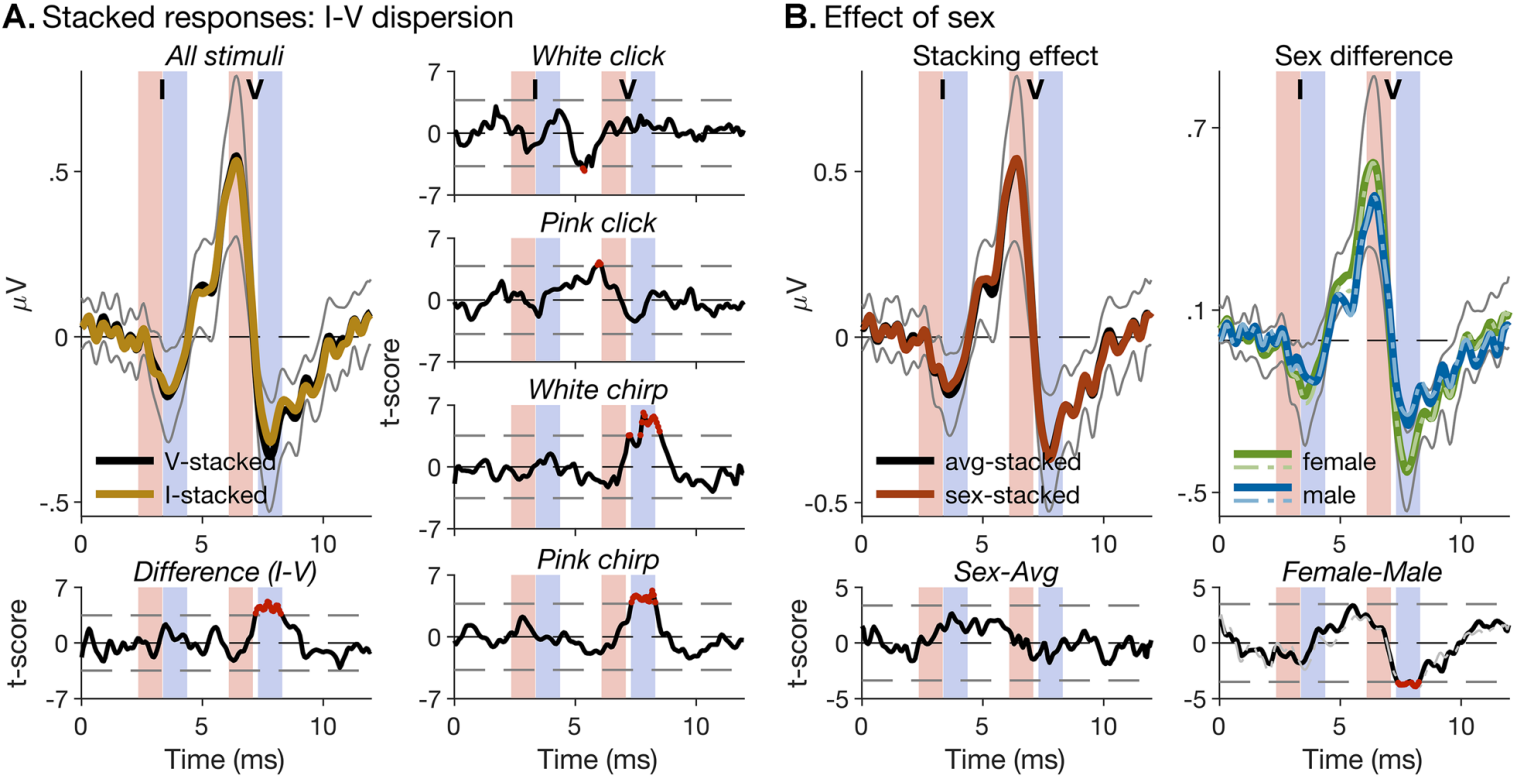
Stacked ABRs simulating responses to wave- and/or sex-tailored chirps. **A** Left panels: The top panel shows average wave-I- and wave-V-stacked responses across all stimulus conditions (white/pink click/chirp). To facilitate comparison, the responses were temporally aligned (using non-linear curve registration; see “Statistics”) before plotting. The bold lines show the average responses across participants, and the thin grey lines show the 95% CI of the wave-V-stacked response to indicate the degree of response variability. The vertical pink- and blue-shaded patches highlight the time ranges of the wave-I and -V peaks and troughs. The bottom panel shows the *t*-scores for the difference between the two responses (see “Statistics”). Significant differences are indicated by red dots. The panels on the right show the *t*-scores for individual stimulus conditions separately. **B** Left panels: same as in **A**, but, in this case, the comparison is between responses stacked either on the average latencies across all participants, or on the average latencies for the relevant sex (see ABR Analysis). Only the average responses across all stimulus conditions are shown. Right panels: average-stacked (thick, solid lines) and sex-specifically stacked (thinner, dash-dotted lines) responses for male and female participants (top) and corresponding *t*-scores (bottom). The amplitude difference between male and female responses appears unchanged by the stacking method

Using stacked ABRs, we can simulate the effect that compensating for wave- or sex-related differences in latency dispersion may have on the broadband wave-I amplitude. Figure 6A shows the comparison, using pointwise one-sample permutation *t*-tests (see “Statistics”), between ABRs stacked on the average wave-V, and the average wave-I latencies, across participants. Stacking on the wave-V latencies mimics the effect of using a wave-V-optimized chirp, like the CE chirp, whilst stacking on the wave-I latencies mimics the effect of a chirp optimized specifically for wave I. The left panels in Fig. 6A show that, in the average response across all stimulus conditions (white/pink click/chirp), stacking on the wave-V latencies produced a small but significant increase in the depth of the wave-V trough. The right panels show that this was mainly driven by the chirp responses (both white and pink). Conversely, however, stacking on the wave-I latencies created no significant increases in either wave-I peak height or trough depth, suggesting that wave-I-optimized chirps would be unlikely to create more reliable wave-I benefit.

Figure 6B suggests an even less convincing case for using sex-optimized chirps, as ABRs stacked on the average latencies for the relevant sex (males on the average male latencies, females on the average female latencies) were not significantly different from ABRs stacked on the overall-average latencies. This was true both for the average responses across stimulus conditions (Fig. 6B, left panels) and for each condition separately (data not shown). At the same time, however, there was a consistent sex difference in stacked ABR amplitudes, with larger female than male amplitudes (Fig. 6B, right panels). The difference was particularly clear in the wave-V time range and was similar between the average- and sex-specifically stacked responses.

### Sex Differences in Broadband ABR Amplitude Originate in Apical Cochlear Regions

**Fig. 7:**
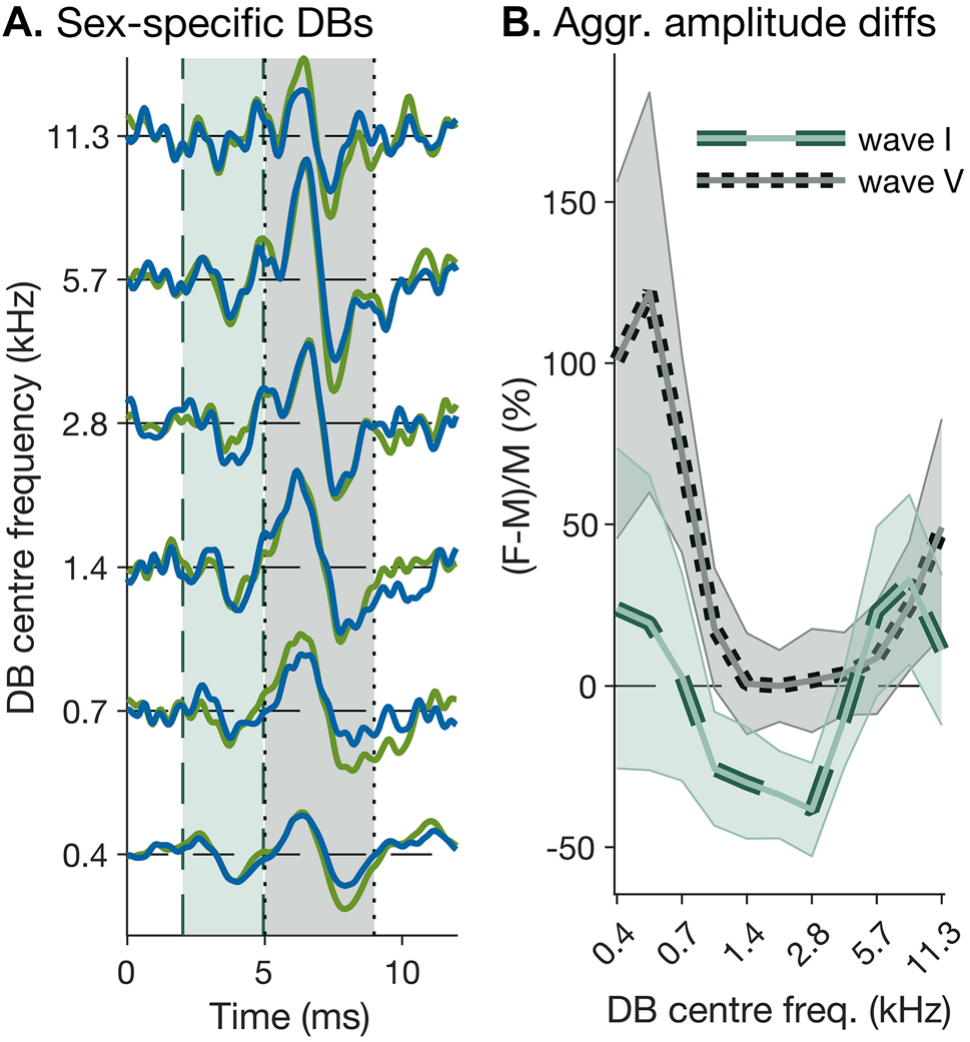
Sex differences in derived-band ABR amplitudes. **A** Average derived-band responses for male and female participants, averaged over all stimulus conditions. Like the stacked responses shown in Fig. 6, the responses were temporally aligned before plotting. The vertical green- and grey-shaded patches show the wave-I and -V time ranges used for calculating the aggregate response amplitudes used in **B** (2–5 and 5–9 ms; see “Statistics”). (B) Aggregate wave-I and -V amplitude differences between female and male derived-band responses, plotted as a function of band centre frequency. The differences are expressed as percentages of the respective male amplitudes (with positive values indicating larger female responses). The bold lines show the average over 5000 bootstrap samples and the semi-transparent patches show the bootstrap standard error. For display purposes, the responses were interpolated to a half-octave spacing using modified Akima interpolation (Akima, 1970) before calculating their aggregate amplitudes. The statistical analysis was based on the uninterpolated responses

Stacked ABRs compensate for latency dispersion *across* derived frequency bands but cannot compensate for dispersion within bands. Sex-related difference in cochlear dispersion may be too small to manifest directly as differences in derived-band latencies but instead could manifest indirectly as differences in derived-band *amplitudes*. Derived-band amplitudes will, of course, also be affected by other, more general, factors, such as skull thickness or brain-to-scalp conductivity (Chauveau et al. 2004; Wang and Ren 2013). These factors are independent of derived-band frequency and should thus affect all derived bands similarly. In contrast, any within-band effects of latency dispersion difference should be limited to the lowest-frequency bands, where dispersion is generally greatest. In line with the latter prediction, the amplitudes of the two lowest-frequency derived bands (0.4 and 0.7 kHz) both showed substantial sex differences (Fig. 7), with larger amplitudes for females than males, particularly for wave V (*t*(65.7) = 2.05, *p* = 0.045). In contrast, the two intermediate bands (1.4 and 2.8 kHz) showed no significant sex differences (in fact, wave I show a marginally significant difference in reverse direction; *t*(58.9) =  − 1.69, *p* = 0.096). The highest two bands (5.7 and 11.3 kHz) showed slightly (but not significantly) larger amplitudes for females than males, similar to the lowest two bands, but these differences may be a trivial consequence of the (also non-significant) difference in high-frequency hearing sensitivity between our male and female participants (see “Participants” and Fig. 2A). Sex differences in derived-band amplitudes were generally greater in wave V than wave I [χ^2^(1) = 8.33, *p* = 0.004, BF_10_ = 2.91]. Whilst their variation across frequencies was statistically weak, reaching only marginal significance (sex-by-frequency interaction: *χ*^2^(5) = 9.38, *p* = 0.095, BF_01_ > 150), its shape was consistent across waves (three-way interaction between sex, frequency and waves: *χ*^2^(10) = 11.5, *p* = 0.323, BF_01_ > 150).

### Individual Stacking Significantly Increases the Effective wave-I Amplitude

**Fig. 8:**
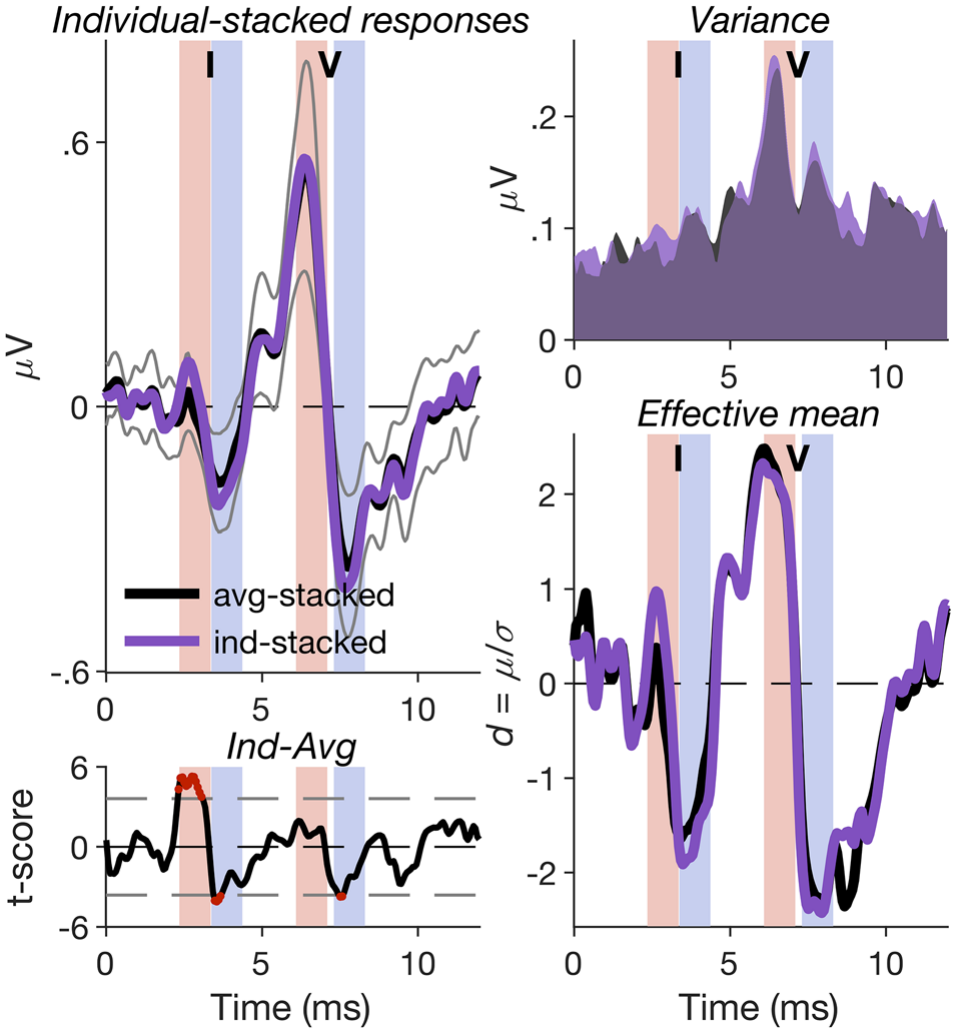
Individual-stacked ABRs. Left panels: comparison between individual- and average-stacked ABRs, averaged across stimulus conditions and plotted as in Fig. 6. The average-stacked ABR was replotted from Fig. 6B. Right panels: The top panel shows the point-wise standard deviation of the average- and individual-stacked ABRs shown in **A**. The bottom panel shows the corresponding effective mean responses (average responses divided by corresponding point-wise standard deviations)

Anatomical results suggest that inter-individual variation in cochlear length exceeds the average sex-related cochlear length difference by a factor of about two (a meta-analysis by Miller 2007, estimated an average sex-related difference of 3.36%, and an inter-individual variance of 6.86%). Compensating for inter-individual differences in ABR latency dispersion may thus afford much greater benefit than compensating for sex-related dispersion difference. Figure 8, which compares individual- and average-stacked ABRs, suggests that individual dispersion compensation may be particularly beneficial for wave I, as wave I showed significantly larger individual- than average-stacked peak and trough amplitudes (left panels). As there was no commensurate increase in inter-individual variance (top right panel), the resulting increase in the “effective mean amplitude” (pointwise average amplitude divided by pointwise standard deviation) of wave I was substantial (bottom right panel). The benefit for wave V was considerably smaller, presumably, because wave V is overall longer and slower (Kevanishvili and Aphonchenko 1979), and thus less affected by inter-individual latency variation than wave I (Petoe et al. 2010).

### Chirps Can Cause Substantial Reduction in Higher-Frequency Derived-Band ABR Amplitudes

**Fig. 9:**
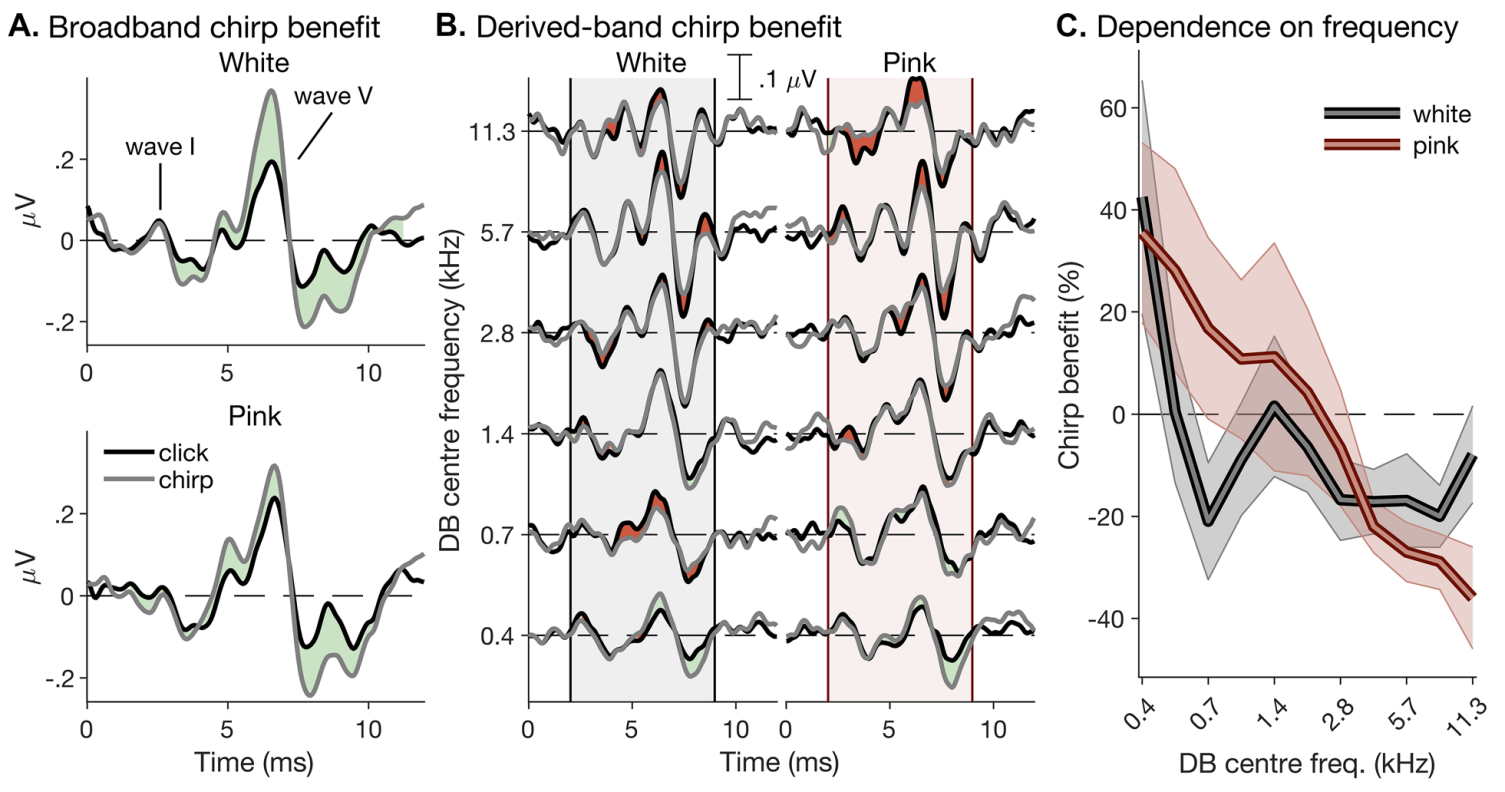
Chirp effect on derived-band responses. **A** Average click- and chirp-evoked broadband ABRs across participants (black and grey lines), plotted separately for white and pink stimulus spectral profiles (top and bottom panels). Like the stacked and derived-band responses shown in Figs. 6, 7, and 8, the responses were temporally aligned before plotting. Regions where the chirp responses were reduced relative to the click responses are highlighted in red, and regions where they were enhanced are highlighted in green. **B** Average click- and chirp-evoked derived bands, plotted in the same way as the broadband responses in **A**. The white and pink spectral conditions are shown in the left and right panels, respectively. The vertical grey- or red-shaded patches show the time range used for calculating the derived-band chirp benefit shown in C (2–9 ms; see “Statistics”). **C** Derived-band chirp benefit, defined as difference in aggregate response amplitude between click- and chirp-evoked derived-band responses over the entire response time course expressed as a percentage of the respective click-evoked amplitude. As in Fig. 7, the bold lines show bootstrap averages, and the semi-transparent patches show their bootstrap standard errors

The functions relating the white and pink click-evoked derived-band latencies to frequency accorded similarly well with the group-delay function of the CE chirp (see Fig. 4A). Given that the pink stimuli contained more low-frequency, and less high-frequency energy, than the white stimuli, the chirp benefit on the broadband ABR amplitudes should thus, if anything, have been greater for the pink than white stimuli. Figure 9A shows that the opposite was actually true: whilst the white chirp afforded a substantial increase in broadband response amplitude, particularly in the time range of wave V, and, to a lesser degree, also in the time range of the wave-I trough, the pink chirp afforded a much more modest benefit on wave V, and little or no benefit at all on wave I.

An explanation is suggested by Fig. 9B, which compares the amplitudes of the click- and chirp-evoked derived band responses for each spectral condition. As chirps create response synchronization not only across, but also within derived bands, the chirp-evoked derived-band responses should be as large as, or larger than, the corresponding click-evoked responses (note that the click and chirp stimuli were exactly matched for energy; see “ABR Acquisition”). This was indeed the case for the lowest-frequency (0.4 kHz) derived-band responses, which, for both spectral conditions, showed substantial chirp-induced amplitude enhancement (by 42.5% and 35.5%, respectively; Fig. 9C). Many of the higher-frequency responses, however, were not only not enhanced by the chirp, but were actually *reduced*. For the white stimuli, the reduction was relatively modest and largely similar (amounting to − 4.92%, on average) across all bands from 0.7 kHz upwards, consistent with similar (albeit non-significant) results by Wegner and Dau (2002). For the pink stimuli, the contrast between enhancement and reduction was both greater (with the maximum enhancement and reduction amounting to 35.5% and − 36.0%, respectively) and more gradual, increasing more progressively from the lowest-frequency (0.4 kHz) to the highest-frequency (11.3 kHz) band. Statistically, these effects were reflected in frequentist, but not Bayesian, significant stimulus-by-frequency interactions (white: *χ*^2^(5) = 13.8, *p* = 0.017, but BF_01_ > 150; pink: *χ*^2^(5) = 28.7, *p* < 0.001, but BF_01_ = 9.54).

## Discussion

This study was aimed to test whether measurements of the supra-threshold ABR wave I deflection — for instance, for the assessment of cochlear synaptopathy — could benefit from using tailored chirp stimuli, specifically designed to compensate for the wave-I latency dispersion within male and female subjects separately. The idea that wave- and/or sex-tailored chirps might enhance the small and inter-individually variable wave I arises from previous reports suggesting that ABR latency dispersion differs both between waves (Morimoto et al. 2019) and between sexes (Don et al. 1993, 1994). Contrary to these reports, our results, which were based on a derived-band ABR data set including both click and wave V-optimized (CE) chirp stimuli, suggested no systematic differences in derived-band latency dispersion, neither between waves nor between sexes. Whilst the function relating the derived-band latencies to frequency was slightly more convex (more curved) for wave V than wave I, and for females than males, the overall latency difference between the lowest- and highest-frequency derived bands was by only ~ 0.18 ms shorter for wave I than for wave V, and was actually longer (by ~ 0.2 ms) for females than for males. Consistent with the latency results, neither the wave- nor the sex-specifically stacked ABRs showed any significant enhancement in wave-I amplitude. In contrast, the individual stacking condition, which compensated for inter-individual latency variation in each wave, showed significant and substantial wave-I enhancement. This is consistent with previous results suggesting that random, yet reliable, inter-individual differences are a predominant contributor to ABR latency and amplitude variation (Edwards et al. 1982; Lauter and Loomis 1986, 1988; Munjal et al. 2016; Prendergast et al. 2018; Guest et al. 2019) and further suggests that inter-individual variation in ABR amplitude can be reduced by accounting for inter-individual variation in ABR latencies.

Both sex-related and inter-individual differences in ABR latency dispersion would be expected to be at least in part due to corresponding differences in cochlear length. A meta-analysis of a large set of anatomical data has suggested that inter-individual variation in cochlear length exceeds the average sex-related cochlear length difference by a factor of about two (Miller 2007). This may explain the discrepancies between the current and previous (Don et al. 1993, 1994; Schoonhoven et al. 2001) results on sex-related ABR latency dispersion difference, with Don et al. (1993) finding a substantial and significant difference of 13%, Schoonhoven et al. finding an unspecified smaller, but non-significant difference, and the current results suggesting no, or even reversed, difference. Under the assumption that sex-related difference in ABR latency dispersion has similar effect size to that estimated by Miller (2007), both the current and previous studies would have been underpowered for detecting a dispersion difference and would thus have been likely to show inter-sample discrepancy (Ioannidis 2005; power would have ranged from 22.1% in Schoonhoven et al. 2001, who used 12 female, and 12 male participants, to 35.7% in Don et al. (1994), who used 23 female, and 20 male participants; in the current study, it would be 35.5%).Whilst the current results revealed no systematic sex difference in derived-band latency dispersion, they showed significant and sizeable sex differences in derived-band wave-V latencies, as well as stacked and derived-band ABR amplitudes. The difference in stacked ABR amplitude is consistent with previously reported sex differences in broadband ABR amplitude (Trune et al. 1988; Chan et al. 1988; McFadden et al. 2021). The difference in derived-band wave-V latencies was constant across derived-band frequencies and was not mirrored in wave I, supporting previous suggestions that sex difference in ABR latencies predominantly reflects difference in neuronal conduction delay resulting from the difference in brain volume (Dempsey et al. 1986; Aoyagi et al. 1990). The sex difference in derived-band amplitudes was largely limited to the two lowest-frequency bands (between 0.5 and 1 kHz and below 0.5 kHz), where cochlear dispersion, and thus any sex-related dispersion difference, would have been greatest (Dau 2003). This raises the possibility that sex-related difference in cochlear dispersion was, after all, present in the current participants, but was too small to yield a detectable dispersion difference in derived-band latencies. Alternatively, sex difference in cochlear dispersion may be specific to apical (lower-frequency) cochlear regions. Based on stimulus-frequency otoacoustic emission (SFOAE) delays, Shera et al. (2010) suggested that apical and basal cochlear regions involve different mechanisms of response generation, with different dispersion profiles. If true, sex-related dispersion difference may be specific to the apical response mechanism and thus only affect lower-frequency cochlear responses. This idea is supported by several reports of sex differences in distortion product (DP) OAE delays, most of which were limited to lower frequencies (Kimberley et al. 1993; Moulin and Kemp 1996; Bowman et al. 2000) and by one report of a sex difference in transient-evoked (TE) OAE delay (Bharadwaj et al. 2019), although this was only measured at a single frequency (2 kHz). Further research will be needed to achieve a more quantitative understanding of sex-related cochlear dispersion difference, and to elucidate its relationship with cochlear response mechanisms.

Unlike previous studies, the current study measured derived-band responses to click and chirp stimuli that were exactly matched in overall energy and spectral composition. A comparison of the click- and chirp-evoked responses revealed, firstly, that chirp-evoked responses are associated with greater underlying dispersion than click-evoked responses, and, secondly, that chirps can cause substantial reduction in ABR contributions from basal (higher-frequency) cochlear regions. Both findings raise the possibility that chirps cause a reweighting of cochlear response contributions from basal (higher-frequency) to apical (lower-frequency) cochlear regions. Some degree of reweighting will occur simply as a result of response synchronization, which will benefit the more strongly dispersed apical responses more than the basal responses. Response synchronization explains the observed enhancements in the lower-frequency derived-band amplitudes for chirps but does not explain the reductions in the higher-frequency amplitudes. Instead, the reductions are probably related to the phenomenon of upward spread of excitation, which is also thought to account for the previously reported decrease in chirp benefit with increasing stimulus intensity (Dau et al. 2000; Elberling and Don 2008; Elberling et al. 2010). As cochlear responses to lower-frequency stimulus components travel apically towards their place of resonance, they also stimulate the basal cochlear places they travel through. For clicks, basal responses to higher- and lower-frequency stimulus components, often referred to as “on-” and “off-frequency” responses, will be synchronous, as all frequency components of clicks are synchronous, and cochlear response latencies are determined by the response-generating cochlear place, rather than the stimulating frequency (see, e.g. Bell 2012). For chirps, however, on- and off-frequency responses will be staggered in accordance with the delays of the eliciting frequency components (see Dau 2003, for an illustration of this effect using an auditory model). As a result, the off-frequency responses will be dyssynchronous with both the on-frequency responses and also with each other, and thus the overall response size will be reduced. According to this reasoning, the amount of chirp-induced reduction of a given response from a given cochlear place should thus depend on the relative strengths of the response’ on- and off-frequency components. This is consistent with the current observed differences in both the pattern and the amount of chirp-induced reductions in derived-band amplitudes between pink and white stimulus spectral profiles, and also provides an alternative explanation for the previously reported reduction in chirp benefit, or even chirp detriment, on the broadband wave-I amplitude (Petoe et al. 2010; Rodrigues and Lewis 2012): rather than indicating a difference in dispersion profile between wave I and V, as suggested by Morimoto et al. (2019), the reduced chirp benefit on wave I is more likely related to wave I’s greater reliance on response contributions from basal (higher-frequency) cochlear regions (Don and Eggermont 1978; Eggermont and Don 1980), where off-frequency responses are relatively stronger.

## Summary and Conclusions

It is generally assumed that frequency-specific click- and chirp-evoked ABR contributions are merely time-shifted relative to one another, but otherwise similar, and thus, that the main determinant of chirp benefit on the aggregate (or broadband) ABR amplitude is the degree of cross-frequency response synchronization achieved by the chirp. Assuming that this assumption was true, the current results would suggest that there should be no additional wave-I benefit from using wave-I-, as opposed to wave-V-optimized chirps, but that there could be some benefit from using sex-tailored chirps. Contrary to this assumption, however, the current results also showed that chirps can create substantial reduction in frequency-specific ABR amplitudes, and that this can counteract, or even abolish, any beneficial effect of cross-frequency response synchronization. Within a given frequency (or cochlear) region, the degree of chirp-induced response reduction will depend on the relative strengths of on- versus off-frequency responses, and thus, the overall impact on the aggregate (broadband) ABR amplitude will depend both on the measured wave (with wave I more affected than wave V) and the stimulus spectral composition. Given that on- and off-frequency cochlear responses rely differentially on inner and outer hair cell function, chirp benefit may also vary between individuals.
